# An Account of the Practice of One of the Physicians of the Westminster General Dispensary, and of the Western Dispensary, from the 20th of March to the 20th of April, 1807

**Published:** 1807-05

**Authors:** Samuel Fothergill

**Affiliations:** Southampton Street, Strand


### *An Account of the Practice of one of the Physicians of the Westminster General Dispensary, and of the Western Dispensary, from the 20th of March to the 20th of April,* 1807.

ACUTE DISEASES.

| | | | |
|---|---|---|---|
| Typhus | 3 | Peripneumony | 2 |
| Catarrh | 8 | Hæmoptoe | 4 |
| | | Cynanche Tonsillaris | 3 |

Acute

| | | | |
|---|---|---|---|
| Acute Rheumatism - | 5 | Gastrodynia - - | 4 |
| Hydrocephalus - - | 2 | Pyrosis - - - | 2 |
| Erysipelas - - - | 1 | Icterus - - - | 3 |
| Enteritis - - - | 1 | Diarrhœa - - - | 2 |
| Hooping Cough - | 2 | Dysentery - - | 2 |
| Acute Diseases of Infants | 7 | Dyspepsia - - | 3 |
| CHRONIC DISEASES. | | Hypochondriasis - | 2 |
| Pulmonary Consumption | 4 | Melancholia - - | 1 |
| Scrophula - - | 2 | Worms - - - | 3 |
| Peripneumonia Notha | 3 | Gravel and Dysure - | 4 |
| Asthma - - - | 2 | Hydrothorax - - | 1 |
| Cough and Dyspnœa - | 36 | Ascites and Anasarca | 4 |
| Hoarseness - - | 3 | Marasmus - - | 2 |
| Asthenia - - - | 12 | Cutaneous Diseases - | 6 |
| Chronic Rheumatism | 6 | Menorrhagia - - | 1 |
| Lumbago - - - | 3 | Amenorrhœa - - | 2 |
| Cephalalgia - - | 2 | Dysmenorrhœa - | 1 |
| Paralysis - - - | 3 | Chlorosis - - - | 2 |
| Pleurodyne - - | 2 | Leucorrhœa - - | 2 |

The weather during the last month has continued cold; and the diseases have been very similar to those enumerated in the former Report. Considerable mortality has prevailed amongst middle aged and elderly people; as far as my own observation goes, children have not suffered in an equal degree; probably from their being more carefully guarded from the severity of the season, from being less exposed to the night air, and from their not being enfeebled by the cares and anxiety which operate upon maturer age.

Affections of the head and paralytic attacks have of late occurred very frequently; it is extremely difficult in many of these cases to ascertain the remote causes which sometimes, for a long period, have almost insensibly been operating upon the system. Men engaged in the active duties of life have not time, opportunity, or inclination to mark any slight deviation from health: and it is not till a disease has made considerable progress that they think of medical assistance; and thus many of the most valuable lives are lost to their friends and to society. On the other hand, people who have little to occupy their attention, people whose chief difficulty is to know how to get through a day, and escape from ennui, are not content with observing the approaches of disease, they anticipate them, and imagine symptoms which they do not feel.

Every

Every one is interested in the science of medicine, as conducing to alleviate and to remove some of the most distressing evils which afflict man. Unfortunately, many people not only feel an interest in medicine on this account; they also wish to practice it, without any pretensions whatever, to skill, to experience, or to information. These uneducated practitioners, are, it must be confessed, frequently successful. They combat the first advance of disease, and sometimes put it to flight; but they much oftener suppose their medicines have subdued symptoms which were not present, and acquire reputation for performing wonders, when there was no danger. This gives them confidence in their practice; they join in the absurd cry against medical men, or at least try all their stock of recipes, and exhaust their store of herbs and simples, before they condescend to employ a physician. In villages and country towns this is more particularly the case, and where no regular practitioner is near, is more tolerable. But to trifle with our own health and that of our neighbours, cannot be commendable; and I think it the duty of every man to hold up his public testimony against the mal-administration of medicine; a practice which seems to be increasing, and is not sufficiently contemned by the regularly educated practitioners of medicine, who may safely denounce it, not from interested motives, but from most sincerely and anxiously desiring to promote to the utmost of their power the welfare of the community.

I could bring forward several cases in illustration of the foregoing observations, but will content myself with very briefly mentioning one, which has recently come under my notice. A lady, aged about forty, plethoric, asthmatical, and of sedentary habits, became affected with cough and shortness of breath, and at the same time complained of a sense of fulness in the head with some degree of vertigo. She regarded the cough as the most important symptom, and allayed it with paregoric elixir and some demulcent medicines of her own making up. She confined herself at home, taking no exercise whatever, and in a few days being free from coughing, considered herself as quite recovered, though she was still occasionally affected with giddiness in her head. Soon afterwards she was seized with a paralytic stroke, from which, after being in considerable danger, she is slowly recovering. Now, had she in the first instance, sent for her apothecary; he told me, as he was well acquainted with her constitution and habits, and had anticipated an attack of this kind, he should certainly tainly tainly have bled her, and prescribed cathartics; and thus most probably would have prevented her present serious malady.

SAMUEL FOTHERGILL.

*Southampton Street, Strand, April* 23, 1807.

